# Adapting, restarting, and terminating a randomised control trial for people with cystic fibrosis: Reflections on the impact of the COVID-19 pandemic upon research in a clinical population

**DOI:** 10.1016/j.conctc.2024.101294

**Published:** 2024-03-27

**Authors:** Owen W. Tomlinson, Alan R. Barker, Sarah Denford, Craig A. Williams

**Affiliations:** a Children's Health and Exercise Research Centre, Department of Public Health and Sports Sciences, University of Exeter, Exeter, United Kingdom; b Academic Department of Respiratory Medicine, Royal Devon University Healthcare NHS Foundation Trust, Exeter, United Kingdom; c Department of Clinical and Biomedical Science, University of Exeter, Exeter, United Kingdom; d Health Protection Research Unit in Behavioural Science and Evaluation, University of Bristol, Bristol, United Kingdom

**Keywords:** Randomised control trial, COVID-19, trial modification, Respiratory disease

## Abstract

**Background:**

Habitual physical activity (PA) and exercise form a cornerstone of the management of cystic fibrosis (CF), a genetically inherited pulmonary and digestive condition – whereby telehealth platforms have been proposed as a mechanism to engage remotely people with CF in PA and exercise.

**Methods:**

To test this, in early 2020, the ‘ActivOnline: Physical Activity in Cystic Fibrosis Trial’ (ActiOn PACT) randomised control trial was established to examine whether an online intervention was effective at increasing PA in adolescents and adults with CF.

**Results:**

The emergence of the COVID-19 pandemic in 2020 forced this trial to be paused and modified, with the adoption of online recruitment and remote assessment of outcome measures. Despite such adaptations in accord with frameworks developed by the National Institute for Health Research, this trial failed to recruit and was subsequently terminated.

**Conclusions:**

This article details the authors reflections upon the proposed reasons for lack of recruitment, including improved technology and medications for people with CF, and contextualises this finding in relation to the wider issue of non-reporting of trial results in clinical research.

## 1 Introduction

Cystic fibrosis (CF) is a genetically inherited condition, currently affecting approximately 10,800 people in the United Kingdom (UK) [1]. It primarily manifests as an accumulation of thick mucus in the airways and digestive tract, resulting in chronic infection and inflammation, with declining pulmonary function that eventually results in respiratory failure and premature mortality [2].

The role of physical activity (PA) and exercise in managing CF is well documented, whereby increased PA can offset declines in lung function [3], is associated with greater quality of life [4], and reduced number of hospitalisations [5], and enhanced long term outcomes [6]. Therefore, regular participation in PA and exercise is recommended for people with CF [7,8]. Despite recommendations, the frequency and duration of participation in PA and exercise is variable [9], with many barriers and facilitators reported in this population [10].

To address this variability in PA, innovative strategies are required to promote PA and exercise. Telehealth has been developed as a feasible method of engaging people with chronic disease remotely [11], although the optimal platforms and modalities in which to engage people in PA have yet to be established.

## 2 The ActiOn PACT study

The ActiOn PACT (ActivOnline: Physical Activity in Cystic Fibrosis Trial) study was originally developed for people with CF in Australia, to identify the efficacy of a novel, web-based intervention –‘ActivOnline’ – in increasing PA in people with CF [12]. The initial feasibility study was deemed to be successful, because of perceived acceptance of the platform by intended users [12], and therefore a full randomised control trial (RCT) was developed to identify the true effect of the intervention. This follow-up RCT was registered on the Australian and New Zealand Clinical Trials Registry on July 13, 2017 (ACTRN12617001009303) [13], and the protocol published in 2019 [14]. The final results of the Australian arm of the trial are now available and indicate that the intervention was no more effective than usual care at promoting PA [15]. It was suggested that the lack of effect may be due to high baseline levels of PA in this cohort, and a limited engagement with the platform [15].

Following this trial registration, a replication RCT was also established in the UK, to determine the effect of this platform in a different country. Based on initial findings from the trial in Australia [12], a small series of modifications to intended target and outcome measures were made by the steering group. These changes were made to widen recruitment and facilitate ease of completion of measures, and included changes to inclusion criteria, removing the need to be treated with intravenous antibiotics, therefore examining the intervention in patients who were not experiencing an exacerbation.

The UK arm of the trial was registered on *ClinicalTrials.gov* (#NCT04249999) [16] and published online on January 31, 2020. Ethics approval was sought from the Health Research Authority (HRA), with an application being submitted on February 14, 2020. A meeting with the South West (Cornwall & Plymouth) Research Ethics Committee (REC) was booked for March 17, 2020, which was held remotely via telephone. Final ethics approval was granted on May 7, 2020 (IRAS: 252371; REC: 20/SW/0048).

## 3 Effects of SARS-CoV-2 pandemic

In early 2020, emergence of the SARS-CoV-2 virus that causes COVID-19 infection, began to emerge, threatening delivery of clinical trials worldwide [17]. Due to the vulnerability of people with CF, trials involving this population were particularly adversely affected [18], as individuals with chronic respiratory disease were considered an ‘at risk’ population and at elevated risk of severe COVID-19 infection [19].

Initial evidence to emerge from the pandemic indicated that people with CF were not as adversely affected as initially presumed, whereby the disease course and outcomes associated with COVID-19 infection did not appear to be notably different to the general population [20]. This may be due to patients being socially accustomed to self-isolation and ‘shielding’ when unwell [20], as well as being physiologically accustomed to cytokine dysfunction and hyper-inflammation associated with COVID-19 [21].

However, despite the low observed impact in people with CF in relation to infection, the pandemic still presented considerable risk towards this population. People with CF displayed increased risk perceptions and safety behaviours regarding COVID-19 infection [22], and shifted to online healthcare as a precautionary measure [23]. Despite this, people with CF exhibited similar anxiety and depressive symptoms as the general population [22]. Moreover, PA was shown to decrease in people with CF due to the closure of facilities and a lack of motivation [24]; a decline that was sustained for over a year after the pandemic first emerged [25].

At the same time as these deleterious impacts upon patients, there were simultaneous changes in healthcare management, such as transitions in delivery of CF services to an online model [26] and reallocation of clinical staff within hospitals [27]. This meant included pausing and adapting research studies [28], and prioritisation of COVID-19 oriented trials [29,30]. Consequently, non-essential trials such as the ActiOn PACT Study were not in a position to begin recruitment, especially in their anticipated in-hospital format. Therefore, the trial was temporarily suspended from May–December 2020, before officially resuming in January 2021.

## 4 Revision and adaptation of trial protocol

In order to adapt aforementioned changes to trial delivery in the wake of the COVID-19 pandemic, a revised protocol for the ActiOn PACT Study was developed. This included changes to recruitment and consenting of participants, as well as obtaining outcome measures remotely without the need for hospital visits. The changes were designed by the research team, in consultation with institutional research governance, and approved by study sponsors. Final ethics approval for amendments was granted by a sub-committee of the original REC on May 4, 2021.

### 4.1 Recruitment

To mitigate against the requirement to recruit directly from clinics because attendance for people with CF was being discouraged [31], an online strategy was employed. This included advertising on social media accounts operated by the research team, and affiliated parties such as charities, professional networks, and university collaborators.

If potential participants were interested, they were directed to an online form (Microsoft Forms; Microsoft, Redmond, USA) to: a) download the participant information sheet, detailing the study, and b) provide contact details for the research team, so that consent/assent forms may be provided. These ‘expression of interest’ forms were aimed at age-appropriate groups (adults, young people, and parents/guardians), with age-appropriate participant information sheets (16+ y, 12–15 y, parent/guardian) available.

Moreover, use of the Cystic Fibrosis Trust ‘Trials Tracker’ – an online clinical trials ‘watchlist’ for patients – was utilised, with the trial being advertised on the platform, which included a dedicated webpage to the trial [32]. Interested participants provide their email address through a submission form, for the research team to make contact, discuss the project further, and organise consent/testing as applicable.

### 4.2 Consent/assent

If participants expressed interest in the study, by providing recruitment details, the researchers would then provide formal letters of invitation and again provide links to online versions of participant information sheets that detail the study and its requirements, as well as associated risks and benefits.

If participants confirmed their desire to be involved, they would then provide informed consent prior to their involvement in this study. This procedure was to be performed electronically, using a secure form hosted by the sponsoring institution, whereby participants stated their name, checked a series of tick-boxes declaring they had downloaded, read, and understood the participant information sheet, and right to withdraw, and declared their consent. The date of consent was automatically collated via this system. Where participants were under the age of 16 y, they were to provide informed assent and their parent/guardian also provided electronic, informed consent on their behalf. Once participants had consented, a copy was generated and sent to participants for them to retain.

This online approach aligns with requirements jointly published by the HRA and Medicines and Healthcare Products Regulatory Agency for documenting consent using electronic methods in studies that do not investigate medicinal products (i.e. non-CTIMP [Clinical Trial of an Investigational Medicinal Product] studies) [33], and therefore does not require any unique or additional ethical oversight.

### 4.3 Outcome measures

Participants recruited via this online pathway were scheduled to perform all measurements in their own home. Questionnaires and PA monitors were to be posted to participants home address, with a pre-paid envelope supplied so that these can be returned at no cost to the participant.

Many participants possess their own spirometers for home-monitoring due to a technology rollout from NHS England [34], and therefore no visits to hospital will have been required to perform these measures. Performing spirometry at home is common for the CF community [35], whereby quality of manoeuvres is shown to be equally acceptable as those in hospital clinics, in both children [36] and adults [37] with CF, whereby >90% of people perform ‘acceptable’ tests per existing guidelines [38] from the American Thoracic Society and European Respiratory Society. Moreover, home spirometry is valued by clinicians for early detection of exacerbations [39]. If participants were performing lung function at home, then the make and model of their home spirometer was noted. If home spirometry was not available for any reason, then data from a recent clinical visit would suffice if the participant can recall this. If these data are not known, or cannot be obtained, then this particular lung function value would be excluded, at no detriment to the participants inclusion (as this is only a secondary outcome and will not adversely affect primary analyses).

Qualitative interviews were scheduled to take place to obtain perceptions on general participation in PA, barriers and facilitators to PA, and factors related to the platform (features, engagement, and suggestions for improvement). These were to be undertaken using freely available video-conferencing software (Zoom; Zoom Video Communications, San Jose, USA), with the participants in their own home. This format also provides the option of directly recording the interview to facilitate transcription at a later time, and have been shown to produce similar word counts and interview lengths as in-person interviews [40].

## 5 Termination of study

These aforementioned adaptations to the trial were implemented to reduce prospective burden and risk upon study participants, being collaboratively designed as a study team, alongside engagement with stakeholders and with approval from study sponsors. This was in accordance with the National Institute for Health Research (NIHR) ‘Restart Framework’ [41].

Continued attempts were made to recruit, including: 1) sustained social media campaigns across multiple accounts using dedicated profiles for the study; 2) liaison with sponsors for assistance with advertising; and communication with academics, and 3) clinicians regarding strategies and efficacy of online recruitment. However, despite changes and the continued efforts of the team, the trial failed to recruit at all (i.e., zero enrolment), and sponsors were notified in September 2021 of intention to close the trial.

Despite the disappointing outcome, both sponsors and funders were supportive of the decision to terminate the trial. However, to mitigate loss of research time and funds, the funders approved a new survey study (distributed online) that examined the effects of the pandemic upon 10.13039/100006131PA in this group. This further work was well received by the CF community, eliciting 156 responses. The data showing that 37% of respondents perceived exercise to “more important” after the pandemic rather than beforehand, in contrast to 13% who perceived it to be “less important” [42].

## 6 Challenges to study

Whilst no exact reasons could be directly determined as to why the trial failed to recruit, two main factors were believed to be responsible.

Firstly, an increase in home-based exercise during continued ‘lockdowns’, gym closures, and suspension of hospital services during the COVID-19 pandemic was believed to be providing direct competition with the online platform intervention. During these periods of ‘lockdown’ and isolation, specific recommendations were made for home-based training [43], and online searches for ‘home based exercise’ increased notably during this time [44], likely reflecting individuals to adapt to this unique and unprecedented time. This likely widespread societal change is supported by surveying of respiratory patients in the UK who indicated that ∼80% of those with chronic respiratory disease were able to undertake some exercise at home during ‘lockdowns’ [45]. Moreover, online exercise classes offer time-flexibility, and 61% of self-reported ‘inactive’ people used online classes during ‘lockdowns’ [46], with CF service also offering online exercise classes for patients [26]. Therefore, it is feasible that our intended online intervention ironically fell victim to the increased volume of telehealth-based PA and exercise interventions that we set out to further evaluate.

Secondly, the widespread introduction of cystic fibrosis transmembrane conductance regulator (CFTR) modulator therapy – drugs that restore function to the underlying defects in the cell – have had a significant impact upon people with mild, moderate, and advanced CF [47]. These medications significantly improve lung function and quality of life [48], with limited evidence also indicating improved exercise tolerance and PA [49], and possible improvements in life expectancy [50]. Therefore, improving access to these medications has been a priority for the wider CF community, and in June 2020, the UK Government announced a commercial arrangement that provided access to elexacaftor/tezacaftor/ivacaftor (‘Kaftrio®’ in Europe; ‘Trikafta’® in the USA), a triple-therapy modulator consisting of three different compounds that can be used by up 90% of people with CF [51]. As a result, the number of people taking Kaftrio® increased from 157 (mainly on compassionate grounds) in January 2020 [52], to 5321 in December 2021 [1]. Therefore, this increased availability of medication has likely led to decreased participation in clinical trials (particularly against the backdrop of COVID-19), and whilst this assumption cannot be wholly verified, it is likely people with CF would rather take this medication (that can positively affect lung function and quality of life [48]) rather than participate in a trial that *may* only improve such clinical outcomes.

## 7 Discussion

This commentary has described, adaptations made to an RCT designed to improve physical activity, in relation to the sudden and unanticipated impact of the COVID-19 pandemic. The lack of recruitment experienced by the trial may have been directly impacted by improved technological and medical access for people with CF, in addition to the indirect and direct consequences of the pandemic itself.

### 7.1 Trial adaptation

This trial became an unfortunate victim of the unprecedented effects of the COVID-19 pandemic, and timelines associated with national restrictions in the UK. As shown by dates of trial registration and ethical meetings, the REC meeting of March 17, 2020 came the day after the UK Government advised against all non-essential contact and travel [53], signalling the start of the first national ‘lockdown’ in the UK. Whilst the continued impact of the pandemic could not be anticipated at the time, nor the continued impact upon clinical trials [54] it was logical to proceed with the intended ethics review and protocol at the time in March 2020, with adaptation to occur if deemed necessary. As it transpired, such changes were required, and resources such as the NHS ‘Restart Framework’ [41] were instrumental in guiding adaptations of the trial.

Changes to the trial included transition to remote recruitment [55] and consenting [33], remote data collection via mail distribution of questionnaires and accelerometers, collation of home spirometry data [35], and virtual interviews. Collectively, these changes were designed to ensure the continued viability of the study, and simultaneously reduce burden upon participants and maintain participant safety by ensuring a clinically vulnerable group could participate in the study without visiting hospitals. However, this did not transpire and for reasons that are unclear. Despite this, the outlined changes of themselves are still worthy for consideration in the design of future studies.

We acknowledge that there has been improved access to online exercise classes [26,46] and activity tracking apps and devices [56], alongside increased experiences and expectations of such technology [57]. This, coupled with the slow pace of adoption of e-technologies into clinical trials [58], but faster development and adoption of the technology into everyday life, has likely meant that the proposed platform (ActivOnline) has become prematurely dated and possibly unattractive as a potential intervention. Moreover, access to medicines that simultaneously improve several health outcomes [48] has likely reduced the disease burden in people with CF, to the extent that participation in such technological trials may be unnecessary.

We note that this was not the only trial that adapted methods due to the pandemic, whereby other studies have also reported changes, predominantly surrounding remote recruitment and data collection like the present trial [59,60]. Whilst the enthusiasm and dedication of research teams to continue their projects cannot be faulted, there remains barriers and facilitators to implementation of remote trials for both researchers and participants [61].

The 10.13039/501100000272NIHR Remote Trial Delivery Working Group identified that prospective research participants may be discouraged by their own digital competency, a perceived lack of support from researchers, and perceived lack of value in remote trials, all things that may counter the flexibility associated with remote trials (no travel costs, reduced COVID risk, less disruption to daily routines) [61]. Moreover, research staff face infrastructure and resource challenges [61], and the advantages and disadvantages of technology, and how information is delivered in recruitment process, have both been identified as priorities by the PRioRiTy Priority Setting Partnership (PSP), in conjunction with the James Lind Alliance [62]. Therefore, continued work to establish truly effective recruitment and implementation of remote trials is warranted.

### 7.2 Telehealth and physical activity

In addition to considering how to effectively recruit to clinical trials in general, it must also be recognised that the interaction of telehealth and PA has presented unique challenges in this field. The former is a rapidly developing and novel field, whereby the latter can be a difficult behavioural intervention to successfully implement.

A recent systematic review has identified various functional features and characteristics of telerehabilitation platforms used in the management of chronic disease [63], with no singular ‘optimal’ platform or method yet to be truly established. As such, this variance in platform designs, alongside aforementioned expectations of such technology [57] may contribute to the final result of the original ActiOn PACT trial in Australia (which the UK trial set out to replicate) being a null finding, mostly due to high baseline activity status, and poor platform engagement [15]. Whilst a disappointing final result for the exceptional time and effort placed into the running of a RCT, this also remains an important finding, and helps guide the CF community towards finding ways of engaging people with CF in PA and exercise, and how to improve these trials for improvement of health status, particularly in hard-to-reach clinical groups. Identifying these optimal modes (should they even exist) has been identified as a focal point by researchers and the wider CF community [64], and therefore, the present work can still contribute towards this research field, despite the issues described herein.

In addition to the challenges associated with telehealth, implementing PA interventions can also prove difficult as PA is a highly complex behaviour, whereby multiple aspects of intervention delivery, recruitment and retention must be considered in trials [65]. Previous studies to assess non-compliance (or non-enrolment) to PA studies have revealed several reasons as to why individuals decline to participate in such interventions, including internal, external, and trial based factors [66].

More specifically, this can include perceiving PA as a low priority and low self-efficacy [67], issues with technology and platforms (for e-health interventions) [68], and the presence of existing medical conditions [69]. However, a lack of time and conflict with existing personal and professional commitments is commonly reported across all studies for lack of enrolment into such interventions [[66], [67], [68], [69]], and directly corroborates existing survey data from people with CF, which states that lack of time (alongside tiredness and illness) is a predominant barrier to PA participation [10]. Therefore, researchers must also consider the unfortunate proposition that PA trials are unattractive to people with CF. This suggestion can be supported by the low participation observed in 10.13039/100006131PA and exercise trials, such as the Australian ActiOn PACT trial (61% of people assessed for eligibility declined to participate) [15], and the ACTIVATE-CF trial (successfully recruited only 40% of their intended sample) [70].

### 7.3 Trial reporting

Finally, whilst the lack of recruitment to this current trial is disappointing, we must acknowledge that there are important lessons still to be learnt. Past analyses indicate 25–30% of RCTs prior to the pandemic were discontinued [71], with poor recruitment cited as a predominant reason [72]. The under-reporting of research has been of international concern for decades [73], and it has also been reported that ∼45% of clinical trials in pulmonary medicine remain unreported after 5 years of study completion [74], alongside 20–30% of trials in other clinical groups (cardiac, cancer etc.) [75,76]. It has been noted that early phase trials are less likely to be reported [74], but this does not mean RCTs are immune from lack of reporting.

To not report results (regardless of positive or negative results) is poor scientific practice, and even unethical for recruited participants, whereby their time, effort, and data is effectively wasted. There is an international mandate, supported by the 10.13039/100004423World Health Organization and signed by groups such as the 10.13039/501100000272NIHR, 10.13039/100010269Wellcome Trust, EU Commission, and 10.13039/501100000265Medical Research Council, that advocates for publishing of trials within 12 months of completion [77]. Whilst this trial was not supported by these organisations, we sought to publish this current report to detail the trial adaptations and theorised explanations for poor recruitment, to therefore continue to adhere to good scientific and clinical trial principles, whilst also aiding future groups and trials to learn from these results.

## 8 Summary

This article has discussed the impact of the COVID-19 pandemic upon its role in the closure of a telehealth RCT in a clinical population. The steps taken to adapt in the wake of the pandemic included using further telehealth technologies and embracing this virtual format that the study sought to examine. Although the findings of the study reported a lack of recruitment, by reporting these results, it adheres to international mandates for prompt reporting of clinical trials, and the study will benefit researchers and patients alike for trial design, recruitment and engagement.

## Ethics approval

Ethics approval for the ActiOn PACT UK Trial was originally granted by the South West (Cornwall & Plymouth) Research Ethics Committee and 10.13039/100005622Health Research Authority (IRAS: 252371; REC: 20/SW/0048).

## Consent for publication

No recruitment took place, and therefore no publication of participant data is presented.

## Availability of data

There is no study data to report, and therefore no data is available.

## Funding

This project was funded by the 10.13039/501100000292Cystic Fibrosis Trust, via a Sport England ‘Core Market’ award. Neither funder had any input in the original design of the study nor its adaptations but did give assent to changes in light of ongoing effects of the COVID-19 pandemic. Neither funder had any input into the present manuscript.

## CRediT authorship contribution statement

**Owen W. Tomlinson:** Conceptualization, Writing – original draft, Writing – review & editing. **Alan R. Barker:** Conceptualization, Writing – review & editing. **Sarah Denford:** Conceptualization, Writing – review & editing. **Craig A. Williams:** Conceptualization, Writing – review & editing.

## Declaration of competing interest

The authors declare the following financial interests/personal relationships which may be considered as potential competing interests:

“OWT reports speaker fees for Beam – an online exercise platform – undertaken wholly independently of the current trial.”
